# Can clinical and urodynamic parameters predict the occurrence of neutralizing antibodies in therapy failure of intradetrusor onabotulinumtoxin A injections in patients with spinal cord injury?

**DOI:** 10.1186/s12894-020-00683-6

**Published:** 2020-08-02

**Authors:** Christian Tiburtius, Ralf Böthig, Birgitt Kowald, Sven Hirschfeld, Roland Thietje

**Affiliations:** 1 Centre for Spinal Injuries, Department Neuro-Urology, BG Trauma Hospital Hamburg, Bergedorfer Str. 10 Germany, 21033 Hamburg, Germany; 2 Biomechanical Laboratory, BG Trauma Hospital Hamburg, Hamburg, Germany; 3 Centre for Spinal Injuries, BG Trauma Hospital Hamburg, Hamburg, Germany

**Keywords:** Neurogenic bladder, Spinal cord injury, Botulinum neurotoxin a, Therapy failure, Overactive detrusor function

## Abstract

**Background:**

The aim of the study was to clarify whether clinical and/or urodynamic parameters could be used to infer the probability of neutralizing antibody (NAb) formation as a possible cause of therapy failure (non-response, NR) in patients with neurogenic detrusor overactivity (NDO) due to acquired spinal cord injury/disease (SCI/D) treated with intradetrusor botulinum neurotoxin A (BoNT-A) injections.

**Methods:**

A retrospective chart review was performed of all patients with SCI/D who underwent both intradetrusor onabotulinumtoxin A injections and the determination of neutralizing antibodies against BoNT-A between January 1, 2002, and December 31, 2018. NR was defined as urodynamically confirmed persistent or reappearing NDO.

**Results:**

A total of 2700 BoNT-A injections in 414 patients were ascertained. In 69 patients with primary NR after the first BoNT-A injection (*n* = 6) or with secondary NR after more than one BoNT-A injection (*n* = 63), an antibody analysis was performed. Antibody examination showed 36 (52.2%) negative, 5 (7.2%) borderline and 14 (each 20.3%) each of positive and highly positive values. Subgroup analysis indicated a correlation between NAb formation and the duration of BoNT-A therapy (*p* = 0.015), the mean number of BoNT-A injections (*p* = 0.011) and the time interval between BoNT-A applications (< 7 months, *p* = 0.022). Urodynamic data analysis indicate significant differences with cut-off values of MCC (< 225 ml, *p* = 0.038) and MDP (> 45 cmH_2_O, *p* = 0.040). However, in the regression analysis models, the predictive value for the occurrence of NAb was too low (MCC: ROC AUC 0.62, MDP: ROC AUC 0.52) to distinguish with sufficient certainty between NAb-positive and NAb-negative NR patients.

**Conclusions:**

Despite significant correlations, clinical and urodynamic parameters are only partially suitable for predicting antibody formation against BoNT-A.

## Background

Patients with spinal cord injury/disease (SCI/D) frequently develop neurogenic lower urinary tract dysfunction (NLUTD). Depending on the level of the lesion, up to 95% of patients suffer from neurogenic detrusor overactivity (NDO) [1]. NDO can cause neurogenic urinary incontinence and other adverse events, such as recurrent urinary tract infection, bladder stones, hydronephrosis and vesicoureteral reflux, contributing to a risk of deteriorated renal function and impaired quality of life [2, 3]. Oral antimuscarinics are effective and commonly used as first-line mono- or combination therapy for NDO [4, 5]. Nevertheless, numerous patients discontinue antimuscarinic treatment due to insufficient responses with the consecutive need for higher drug doses or because patients suffer from intolerable side effects such as dry mouth, constipation or sight disorder [6, 7]. Intradetrusor botulinum neurotoxin A (BoNT-A) injections have basically changed the treatment options in patients with refractory NDO and have become a well-established and widely accepted second-line treatment for these patients [8–10]. The European Association of Urology (EAU) Guidelines strongly recommend the use of BoNT-A injection in the detrusor to reduce NDO in SCI/D patients if antimuscarinic therapy is ineffective [3].

Although BoNT-A has been very successfully used for the treatment of refractory NDO for approximately two decades, there is a considerable number of cases of treatment discontinuation and non-response (NR). Recent studies have investigated the long-term outcomes and reasons for the discontinuation of BoNT-A injections [11, 12]. However, the detailed mechanisms of therapy failure are unclear; biochemical processes such as antibody formation and structural changes in the detrusor muscle have been discussed [13], as well as technical issues and catheterization-related difficulties [14]. If neutralizing antibodies (NAbs) against BoNT-A can be detected, they are considered the cause of treatment failure. Nevertheless, only a few studies have investigated the role of NAbs in patients with therapy failure [15, 16], especially in SCI/D patients with NDO.

Recently, various possibilities have been discussed to improve the long-term success rate of BoNT-A therapy. Among others, a change from onabotulinumtoxin A to abobotulinumtoxin A or vice versa has shown certain chances of success [17]. Otherwise, only considerably more invasive surgical treatment options are often considered. In these cases, for individual therapy recommendations, it would be important to know which patients are better suited for each therapy option.

Currently, the question of whether antibody formation is a possible cause of non-response has become particularly important. However, antibody determination is expensive and complex.

It therefore seems important to investigate whether clinical or urodynamic data can predict antibody formation as a possible cause of therapy failure in the case of BoNT-A injection for NDO due to SCI/D.

## Methods

At the Spinal Cord Injury Centre, BG Klinikum Hamburg, Germany, we processed a monocentric retrospective chart review of SCI/D patients treated with BoNT-A injections and searched for patients who underwent single or repeated antibody examinations due to primary (PNR) or secondary (SNR) NR. NR was defined as persistent or reappearing NDO.

Prior to each BoNT-A injection, the preoperative assessment included urinalysis and culture. In the case of positive urine culture, patients received antibiotic treatment according to an antibiogram. Patients with negative urine culture received a single shot of antibiotic prophylaxis with a second-generation cephalosporin. All BoNT-A injections were performed under cystoscopic control by consultants under local or general anaesthesia according to current recommendations [18, 19]. All patients received 200 U or 300 U onabotulinumtoxin A. We only used the newer formulation of the Botox® preparation (Allergan, Inc., Irvine, California) with increased specific activity (i.e., less BoNT protein per unit of activity of 5 ng/100 U). After approval of 200 U Botox® in 2011 for the diagnosis “urinary incontinence in adults with neurogenic detrusor hyperactivity in the neurogenic bladder due to stable subcervical spinal cord injury or multiple sclerosis”, a dose of 200 U was initially started if patients fitted into the approval. Patients mostly received low-dose antimuscarinic therapy accompanied by BoNT-A injections to extend the injection intervals, except when there were intolerable side effects due to antimuscarinic therapy.

For the clinical signs of persistent or reappearing NDO after BoNT-A injection therapy, we evaluated the occurrence of self-reported incontinence episodes between aseptic intermittent catheterization or, with preserved sensitivity, a remarkable increase in catheterization frequency in combination with a decrease in catheterization volume within the first 3 months after BoNT-A injection. All patients with these clinical signs of therapy failure within 3 months after BoNT-A injection (after exclusion or treatment for a causative urinary tract infection (UTI)) were screened for the presence of neutralizing antibodies and received a urodynamic examination for the confirmation of NDO [20] within this period. Antibody tests were performed with a commercially available Mouse Phrenic Nerve Hemidiaphragm Assay for measuring neutralizing antibodies (Toxogen®, Toxogen GmbH, Hannover, Germany). The results of this test were grouped as “negative”, “borderline”, “positive” or “highly positive”. For theoretical and clinical reasons, the analyses of the negative tests were compared with the other groups.

Urodynamic studies were performed according to International Continence Society standards [21, 22] after the exclusion of UTI by urinalysis and culture. We evaluated the following two urodynamic parameters as essential criteria for a lack of effectiveness: maximum cystometric capacity (MCC, in the absence of sensation, the clinician decided to terminate filling in accordance with high detrusor filling pressure due to involuntary detrusor contraction/NDO) and maximum detrusor pressure (MDP) during filling cystometry [23].

The inclusion criteria of the study were as follows: SCI/D, age ≥ 14 years, no previous reconstructive surgery, clinical or “self-reported” signs of PNR or SNR with subsequent urodynamic evaluation and antibody testing after at least one BoNT-A injection due to NDO, independent of any possible antimuscarinic concomitant medication. Further botulinum toxin therapy as a result of non-urological indications, e.g., spasticity, was considered an exclusion criterion.

The data were entered into a database and anonymized during entry. The data are presented as the mean and standard deviation (SD) or as the median with 25 and 75% interquartile ranges (IQRs), depending on the presence of a normal distribution. The statistical significance level was set to *p* < 0.05 for all tests. By means of the Shapiro-Wilk test, the existence of a normal distribution of the data was tested. In the case of two groups with normally distributed data, Student’s t-test was used. For non-normalized data, the Wilcoxon rank sum test was used. Logistic regression analysis was used to investigate which features influenced the likelihood of antibody formation. Fisher’s Chi-square test or Fisher’s exact test was used to show a relationship between qualitative characteristics. Statistical analyses were conducted using SAS 9.2 software (SAS Institute Inc., Cary, NC, USA).

The neurological level of SCI/D was classified according to the ISNCSCI and the severity (degree of impairment) in accordance with the American Spinal Injury Association (ASIA) Impairment Scale (AIS) [24].

The Institutional Review Board (IRB, Hauptgeschäftsführer der Berufsgenossenschaft für Gesundheitsdienst und Wohlfahrtspflege, Pappelallee 33, 22,089 Hamburg) approved the present study, and all applicable institutional and governmental regulations concerning the ethical use of the data were followed.

We certify that all applicable institutional and governmental regulations concerning the ethical use of human volunteers were followed during the course of this research.

## Results

Between January 1, 2002, and December 31, 2018, a total of 2700 BoNT-A injections were performed in 414 SCI/D patients at the Spinal Cord Injury Centre, BG Klinikum Hamburg, Germany. All patients suffered from intolerable side effects or were clinically and urodynamically refractory to primary oral or intravesical antimuscarinic treatment prior to BoNT-A therapy. All patients underwent aseptic intermittent catheterization. Overall, 69 patients (16.7%) met the study inclusion criteria. Table 1 shows the demographic and SCI/D-related characteristics of all 69 NR patients.

**Table 1:** patient’s characteristics

Patient’s characteristics	
No. pts.	414
No. pts. with Non-Response	69
male (%)	53 (77)
female (%)	16 (23)
Tetraplegic n (%)	24 (35)
Paraplegic n (%)	45 (65)
AIS A *n* (%)	49 (71)
AIS B *n* (%)	9 (13)
AIS C *n* (%)	7 (10)
AIS D *n* (%)	4 (6)
C1 – C4, AIS A, B, C *n* (%)	8 (12)
C5– C8, AIS A, B, C *n* (%)	13 (19)
T1 – S5, AIS A, B, C *n* (%)	44 (64)
any level, AIS D *n* (%)	4 (5)
Age at SCI/D (mean; SD; range), years	31.76; 13.99; 4–73
Age at first BoNT-A injection (mean; SD; range), years	39.56; 14.33; 14–85

(*NR* Non response, *AIS* American Spinal Injury Association Impairment Scale, *SCI/D* Spinal cord injury/disease, *BoNT-A* botulinum neurotoxin A)

Among all 69 NR patients, 6 had PNR (8.7%) after the first BoNT-A injection, and 63 had SNR (91.3%). Thus, 91.3% of all 69 NR patients had received more than one onabotulinumtoxin A injection (SNR).

Antibody examination showed the following results: 36/69 (52.2%) patients with clinically and urodynamically confirmed NR had no antibodies, and 14/69 (20.3%) had highly positive and 14/69 (20.3%) had positive antibody levels. A total of 5/69 (7.2%) patients were grouped as borderline (Table 2). A total of 5/6 patients with PNR had no antibody detection, and one patient was classified as borderline. The mean age at non-response was 44.91 years. The median period of time between the date of SCI/D and the first BoNT-A injection was 6.56 years. The median duration between the first BoNT-A injection and NR was 4.91 years. The median interval between BoNT-A injections was 7.85 months. The median number of intradetrusor BoNT-A injections until NR added up to 8.

**Table 2:** Patients with non-response

Patients with Non-Response	***n*** = 69
Primary Non-Response (%)	6 (9)
Secondary Non-Response n (%)	63 (91)
Antibody high positive n (%)	14 (20)
Antibody positive *n* (%)	14 (20)
Antibody borderline *n* (%)	5 (8)
Antibody negative *n* (%)	36 (52)
Age at Non-Response (mean ± SD; range), years	44.91 ± 14.75; 15–86
Time from SCI/D onset to first BoNT-A (median; 25%/75%; range), years	6.57; 2.66/10.84; 0–34
Time from first BoNT-A injection to Non-Response (median; 25%/75%; range), years	4.91; 2.94/7.26; 0–14
Number of BoNT-A injections (median; 25%/75%; range)	8; 4/11; 1–24
Time interval between BoNT-A injections (median; 25%/75%; range), month	7.85; 6.4/8.97; 1–18

(*SCI/D* Spinal cord injury/disease, *BoNT-A* botulinum neurotoxin A)

Of all 414 BoNT-A patients, 28 (6.7%) developed antibodies, 14 (3.4%) with highly positive and 14 (3.4%) with positive levels against onabotulinumtoxin A, including the borderline group (*n* = 5), 7.9% did.

### A – results of demographic and clinical data comparison

To clarify whether it is possible to find correlations between the formation of antibodies against BoNT-A and demographic and clinical data, we statistically examined the following groups of NR patients: patients with highly positive, positive and borderline antibody titres (*n* = 33) versus patients with negative (*n* = 36) antibody titres (Table 3).

**Table 3:** demographic and clinical correlations for antibody formation

	Evaluation	Statistical Test, ***P***-value	Significance
1	Male vs. female	Chi^2^ = 0.710	non-significant
2	PNR vs. SNR	exact Fisher = 0.108	non-significant
3	Motor. + sensor. Complete vs. incomplete lesion (AIS A vs. AIS B, C, D)	Chi^2^ = 0.764	non-significant
4	Motor. complete vs. motor. Incomplete lesion (AIS A, B vs. AIS C, D)	Chi^2^ = 0.406	non-significant
5	Severity of SCI/D	exact Fisher = 0.190	non-significant
6	Age at SCI/D	exact Fisher = 0.257	non-significant
7	Age at first BoNT-A injection	exact Fisher = 0.212	non-significant
8	Age at Non-Response	exact Fisher = 0.529	non-significant
9	Time interval onset SCI/D to first BoNT-A injection	Mann-Whitney-U = 0.788	non-significant
10	Mean time interval first BoNT-A injection to Non-Response	T-Test = 0.015	significant
11	Number of BoNT-A injections Number of BoNT-A injections, Cut off > 5	Mann-Whitney-U = 0.011 Chi^2^ = 0.024	significant significant
12	Median time interval between BoNT-A injections Median time interval between BoNT-A injections, Cut off < 7 month	Mann-Whitney-U = 0.320 Chi^2^ = 0.022	non-significant significant

(*PNR* Primary non response, *SNR* Secondary non response, *AIS* American Spinal Injury Association Impairment Scale, *SCI/D* Spinal cord injury/disease, *BoNT-A* botulinum neurotoxin A)

We found no significant differences between the sexes or between patients with primary or secondary therapy failure. The extent of neurological deficits (senso-motoric complete or incomplete paralysis) and the severity of SCI/D had no significant influence on the probability of antibody formation. Tetraplegics with NR did not produce antibodies more frequently than paraplegics or vice versa. The probability of antibody formation did not differ with respect to the age of the patients at the time of paralysis onset, the first BoNT-A injection or therapy failure. Additionally, the time interval between the onset of paralysis and the first injection of BoNT-A did not influence the probability of antibody formation as the cause of therapy failure.

**Fig. 1:**
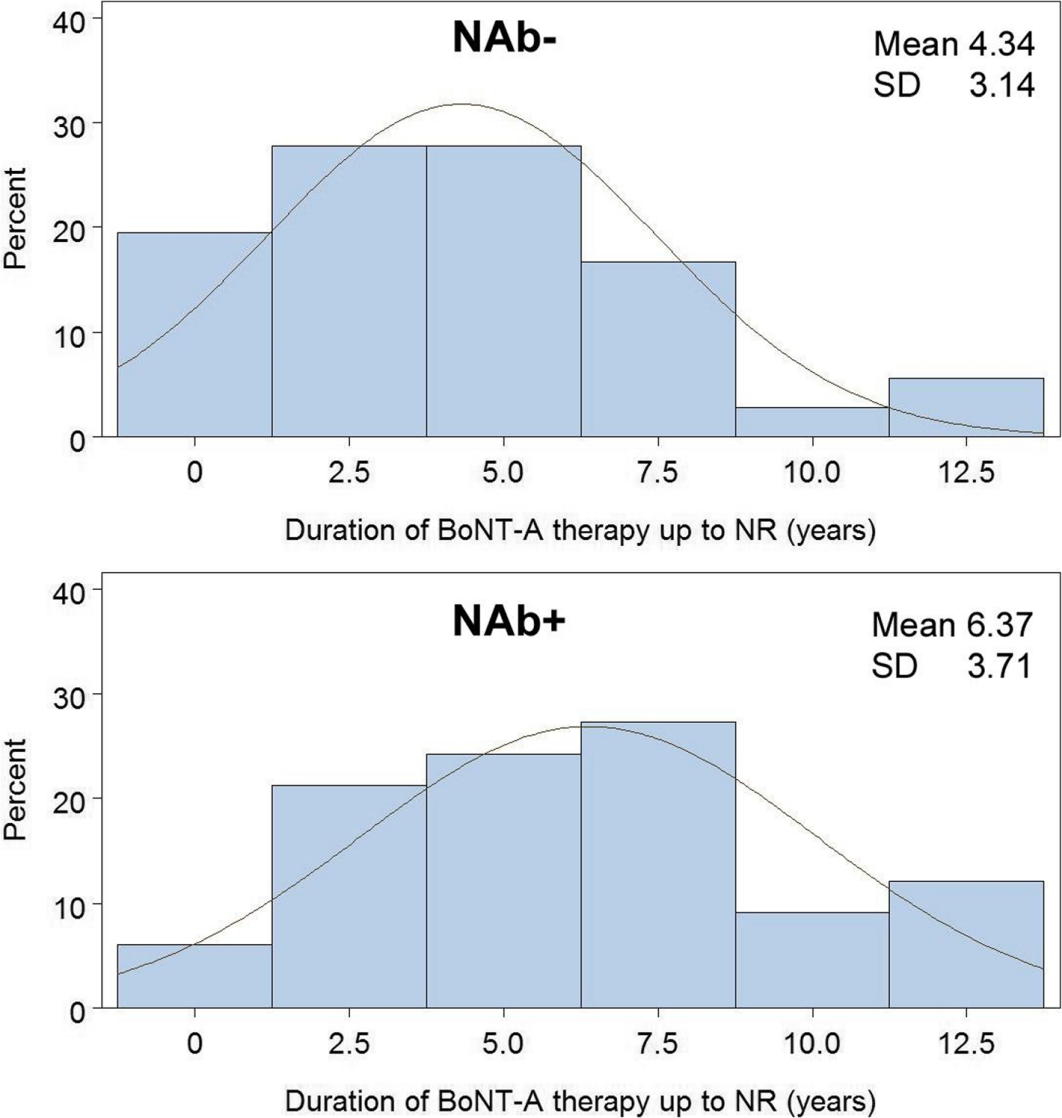
Mean duration of BoNT-A therapy up to the time of NR in NAb+ and Nab patients, *p* = 0.0153

**Fig. 2:**
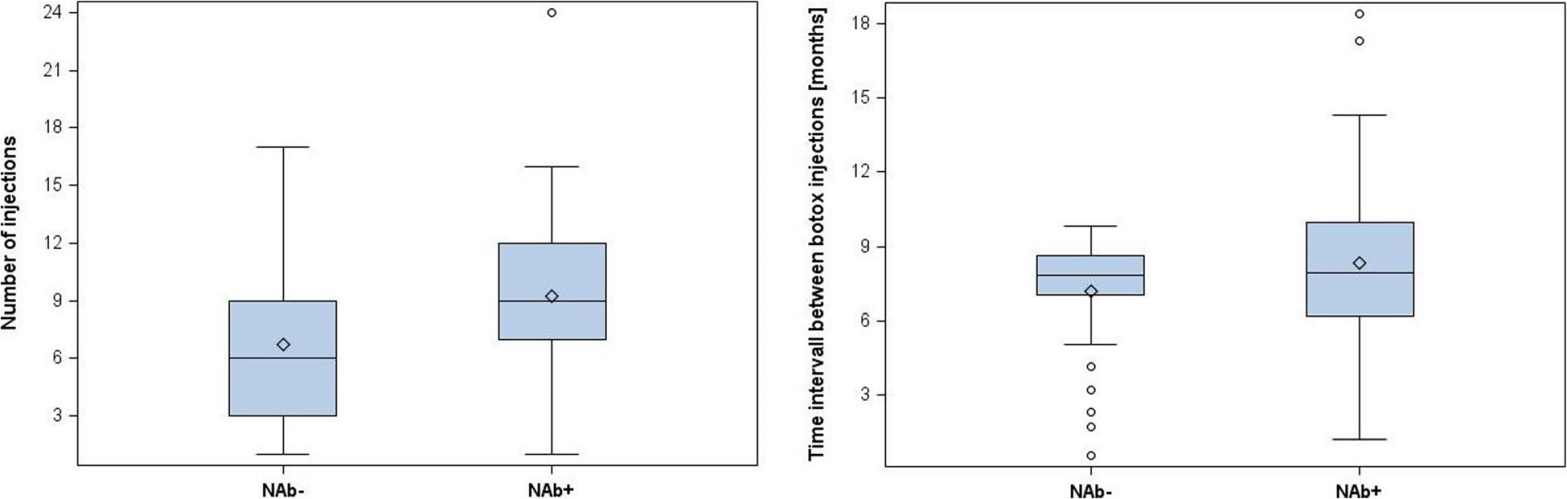
**a** Median number of BoNT-A injections; *p* = 0.0108; **b** Median interval between BoNT-A injections; *p* = 0.3201

On the other hand, with increasing duration of BoNT-A therapy until the occurrence of NR, the probability of NAb formation seemed to increase significantly (NAb neg. vs. pos.: 4.34 years. ± 3.14 vs. 6.37 yrs. ± 3.71; *p* = 0.015, Fig. 1). The median value of the number of BoNT-A injections also differed significantly between NR patients with and without NAb formation (NAB neg. vs. pos.: 6, 3–9 vs. 9 7–12; *p* = 0.011). The probability of antibody formation increased significantly after the 5th BoNT-A injection (Chi^2^, *p* = 0.024, Fig. 2a). The time interval between BoNT-A injections also showed an influence on the probability of NAb formation. Although there was no significant difference between the mean values of the intervals of BoNT-A injections in patients with and without NAb formation (NAB neg. vs. pos.: 7.84 months, 7.03–8.64 vs. 7.91 months, 6.18–9.99; *p* = 0.320), a mean interval between BoNT-A injections of less than 7 months significantly increased the risk of positive antibody detection (Chi^2^, *p* = 0.022, Fig. 2b).

**Fig. 3:**
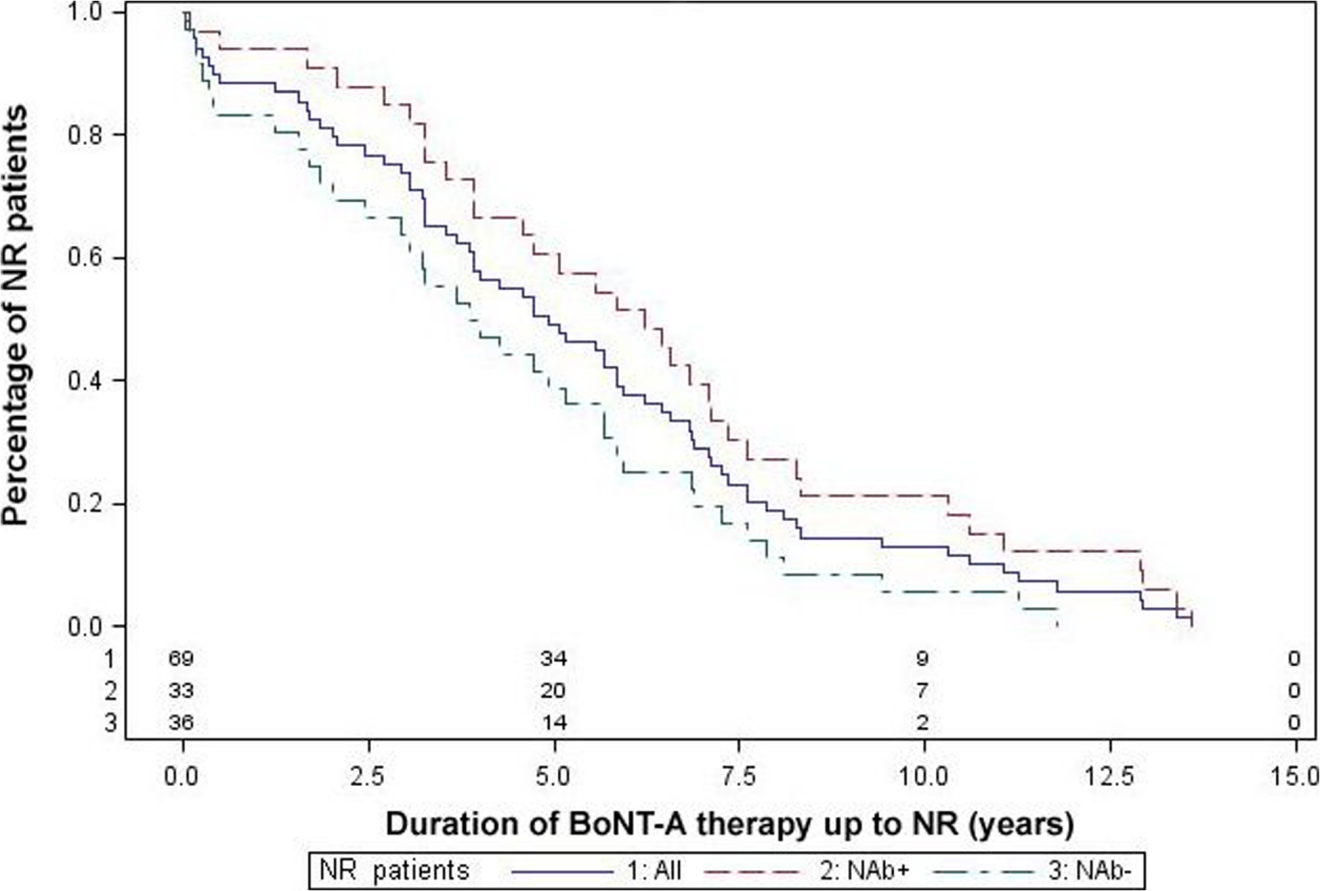
Time course of non-response in NAb+ and NAb- patients

In summary, the data show that the mean number of BoNT-A injections, a cut-off value of more than 5 injections, a longer mean duration of BoNT-A therapy up to non-response and intervals between BoNT-A injections less than 7 months are significantly correlated with a higher probability of antibody formation. This leads to a time shift of therapy failure to the right of the curve in NR patients who produce antibodies (Fig. 3).

**Fig. 4:**
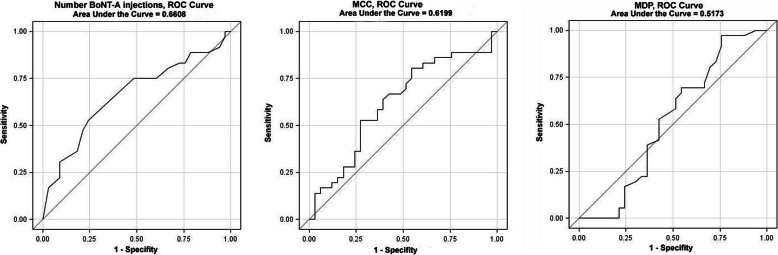
**a** Number of BoNT-A injections, ROC curve, AUC = 0.661; **b** MCC, ROC curve, AUC = 0.612; **c** MDP, ROC curve, AUC = 0.520

However, the cut-off value of the “number of BoNT-A injections” determined by means of an adapted logistic function could only distinguish very poorly between patients with and without antibody formation (ROC AUC 0.66, Fig. 4a).

### B – results of urodynamic data comparison

To confirm the presence of NR, a urodynamic study was performed in each patient with clinical signs of non-response.

For the two urodynamic parameters MCC and MDP, there were no significant differences in the probability of antibody formation in the statistical comparison of the medians (MCC: NAb neg. vs. pos.: 293.5 ml, ± 111.5 vs. 256.6 ml, ±105.8; *p* = 0.164 and MDP: NAb neg. vs. pos.: 31.56 cm H_2_O, ± 9.23 vs. 32.42 cm H_2_O, ± 15.81; *p* = 0.784). However, in subgroup analysis, significantly more frequent antibody formation could be detected with a cut-off value for MCC < = 225 ml (Chi^2^, *p* = 0.038) and for MDP > 45 cmH_2_O (Fisher’s exact test, *p* = 0.040) (Table 4). However, both cut-off values determined by an adapted logistic function could not discriminate with sufficient accuracy between patients with and without antibodies: the ROC AUC for MCC was 0.62 (Fig. 4b), and the ROC AUC for MDP was 0.52 (Fig. 4c).

**Table 4:** urodynamic correlations for antibody formation

Evaluation	mean; SD; range	Statistical Test, ***P***-value	Significance
MCC (ml)	NAb - 293.5; 111.5; 64–468 NAB + 256.6; 105.8; 58–482	t-Test = 0.164	non-significant
MCC cut off <= 225 ml		Chi^2^ = 0.038	significant
MDP (cmH_2_O)	NAB - 31.5556; 9.2256; 15–55 NAb + 32.4242; 15.8055; 7–61	t-Test = 0.784	non-significant
MDP cut off > 45 cmH_2_O		exact Fisher = 0.040	significant

(*MCC* maximum cystometric capacity, *MDP* maximum detrusor pressure)sss

## Discussion

To the best of our knowledge, the present study represents the largest population of SCI patients with therapy failure after BoNT-A injections into the detrusor in which the determination of neutralizing antibodies against onabotulinumtoxin A was performed. Using clinical, demographic and urodynamic data, correlations with the probability of antibody formation were analysed. Although significant correlations were found among the duration of BoNT-A therapy, the number of injections, the mean therapy interval and urodynamic parameters (MCC and MDP), the “adapted logistic function” did not allow the definition of cut-off points that could distinguish with sufficient certainty between NAb-positive and NAb-negative NR patients.

Although BoNT-A injections have been used very successfully and safely for many years for the treatment of refractory NDO in patients with SCI/D [25], there are a significant number of treatment failures and a relatively large number of treatment discontinuations during long-term follow-up. In 2009, Pannek et al. reported an approximate long-term success rate of 74% [26]. In a recent review [27], summarizing 18 studies with a total of 1533 patients, discontinuation rates between 12.1 and 45.2% were described. A 10-year observation period in 140 patients showed an estimated 10-year discontinuation-free survival rate of 49.1% [13] and a discontinuation rate of 40% [12]. In addition to personal decisions and technical problems, antibody formation or structural changes in the detrusor are considered the main cause of treatment discontinuation. Nevertheless, the role of NAbs in therapy failure of refractory NDO has not been ultimately clarified. Until now, only a few studies and limited data have existed regarding the risk of NAb formation in patients with NDO. In previous studies in urological patients, in contrast to striated muscular injection treatments due to spasticity, no antibodies were detected [9, 28]. In contrast, a recent meta-analysis of NAbs in BoNT-A treatment showed the following results [16]: in five studies with data from 294 patients with urological indications, the frequency of NAbs was reported to be 2.7% (95% confidence interval (95% CI) 0.009–0.064); in clinically responding urological patients, the NAb frequency was 3.8% (95% CI 0.011–0.123); and no studies that examined NAbs in SNR patients for urologic indications were identified. In 2008, Schulte-Baukloh et al. [29] reported on NAbs in 8 of 25 patients (4 positives, 4 borderline) after intradetrusor BoNT-A injection, with complete therapy failure in 3 of 4 patients with positive titres. However, the authors found no correlation among the number of BoNT-A injections (*p* = 0.124), the injection interval (*p* = 0.815), or the total amount of BoNT-A applied (*p* = 0.090). Hegele et al. [15] showed NAb formation in 5 patients (16.1%) out of 31 patients treated with intradetrusor BoNT-A injections due to an overactive bladder. In a 3-year, open-label extension study of two 52-week, phase III studies (DIGNITY) in 117 centres in North America, Europe, Latin America, South Africa, and the Asia-Pacific region [30, 31], 8 of 381 patients (all SCI, 2.1%) seroconverted to positive NAbs. Four of these 8 patients continued to experience clinical benefits even after seroconversion. It was striking that the patients with seroconversion had shorter intervals (4.9 months) between BoNT-A injections than the total population (9.1 months).

It should be noted that in all the studies mentioned and in our own patients, only the newer formulation of Botox® with lower antigenicity was used. Allergan changed the composition of Botox® in 1997: the protein content decreased from 25 ng/100 U to 5 ng/100 U. Afterwards, the risk of antibody formation in patients with cervical dystonia decreased by a factor of 6 [32].

In our own 17-year study, we found a total failure rate of 16.7% (69/414), 1.4% with PNR (6/414) and 15.2% with SNR (63/414). Slightly more than half of NR patients did not show NAbs against BoNT-A (52.2%, 36/69), each 20.3% (14/69) showed positive or highly positive antibody levels, and 7.2% (5/69) were grouped as borderline. An analysis of the risk factors for NAb formation in NR showed a significant increase in the probability of positive NAb detection with both an increasing duration of therapy and an increasing number of BoNT-A injections of more than 5 repeated BoNT-A injections. In accordance with this finding, a gradually decreased therapeutic effect after the 4th BoNT-A injection in SCI/D patients with NDO was reported [33]. Furthermore, a significant decrease in quality of life in patients who received five or more injections was described [27].

Perhaps the two risk factors for antibody formation mentioned above describe the same phenomenon: antigenicity that leads to a higher probability of antibody formation as a cause of NR due to the duration of therapy and the number of injections. Our data showed that treatment failure occurred significantly later in patients with Nab formation than in NR patients without NAb formation.

We identified a second risk factor for the formation of NAb in NR with a mean interval between BoNT-A injections < 7 months. This was also shown in a large extension study [31], in which all 8 NAb-positive SCI/D patients had significantly shorter therapy intervals.

Recent studies from France reported a benefit of approximately 50% in patients with intradetrusor injection failure in NDO with botulinum toxin switching from onabotulinumtoxin A to abobotulinumtoxin A or the reverse order [34]. The clinical and urodynamic success rates were 51.7, 57.7 and 56.14% [17, 35, 36]. This corresponds to the proportion of NAb-“non-negative” patients with therapy failure in our study. Unfortunately, no antibody determinations were made in the studies mentioned above. Here, it would be of great interest to look for a correlation between antibody formation and a successful toxin switch.

From a theoretical point of view, it could be assumed that NAb-positive NR patients are more likely to benefit from a toxin change than patients without antibody formation who do not (or no longer) respond to BoNT-A injections because in NAb-positive NR patients, the cause of therapy failure is probably due to the immunogenicity of the BoNT-A preparation. The cause of therapy failure in NAb-negative NR patients is currently not well understood. Histological examinations of the detrusor [37] or the urothelium and suburothelium [38] cannot detect the suspected ultra-structural changes in the bladder wall after (repeated) BoNT-A injections. In contrast, after BoNT-A injections, there was significantly less fibrosis of the bladder wall than in untreated patients. Furthermore, a comparison of samples from patients who responded to BoNT-A therapy and from patients who did not respond showed no difference in inflammation. Responders to BoNT-A therapy tend to show less fibrosis and oedema of the bladder wall than non-responders [39].

For patients who do not benefit from a toxin switch, surgical therapy alternatives must often be considered. It would therefore be valuable if, even without antibody testing, clinical and urodynamic evidence could be used to distinguish between NR patients with and without NAb formation. The identification of NR patients without antibody formation (corresponding to a “negative” result on the NAb test) could be helpful for the clinical decision-making process.

In this context, the present study also examined whether urodynamic parameters are suitable to predict the formation of NAb as a cause of NR. The statistical evaluations show that the probability of positive antibody detection increases significantly in NR patients with low MCC (< 225 ml) and high MDP (> 45 cmH_2_O). Therefore, it seems plausible that patients with smaller MCC and higher MDP values might have early recurrence of NDO and would benefit from an earlier BoNT-A injection than those who without these values. This would be a possible explanation for why these patients produce antibodies more frequently. However, neither these urodynamic nor the clinical criteria presented above are sufficiently successful in our analysis to identify or exclude with sufficient certainty NR patients with antibody formation or to identify NAb-negative NR patients. In this regard, further studies are needed to better understand this potential context.

It is important to recognize that the present study has some limitations. First, this is due to the retrospective nature of data collection with a limited number of participants, but to our knowledge, there is no larger long-term study with NAb determination in the literature. Second, no additional data on concomitant medication, e.g., oral or intravesical antimuscarinics, are available. Finally, no difference was made between injections of 200 or 300 MU onabotulinumtoxin A. These limitations may cause some biases that must be considered in the interpretation of our results. These problems can only be solved by future prospective studies with a strict study protocol and larger study groups. Nevertheless, the authors believe that the presented results may provide some important basic information about possible associations between clinical and urodynamic data and the probability of NAb formation in NLUTD patients with therapy failure after intradetrusor BoNT-A injections.

## Conclusions

The present study shows that despite some significant correlations (duration of BoNT-A therapy, number of BoNT-A injections, interval between injections, and urodynamic parameters such as MCC and MDP), the probability of the occurrence of antibodies against BoNT-A in the case of therapy failure cannot be determined with sufficient certainty using clinical and urodynamic data alone. The predictive value of these parameters is too low to indicate antibody formation as the cause of NR. A recommendation for the subsequent therapy of non-responders to BoNT-A can therefore not be derived from these investigations.

Further studies are necessary to improve the evidence of follow-up therapy in the case of therapy failure against BoNT-A.

## Data Availability

All data generated or analysed during this study are included in this published article. The original data files and statistical analyses are available from the corresponding author upon reasonable request.

## Abbreviations

AIS: American Spinal Injury Association impairment scale;
ASIA: American Spinal Injury Association
AUC: Area under curve
BoNT-A: Botulinum neurotoxin A
CI: Confidence interval
IQR: Interquartile range
MCC: Maximum cystometric capacity
MDP: Maximum detrusor pressure
NAb: Neutralizing antibodies;
NDO: Neurogenic detrusor overactivity
NLUTD: Neurogenic lower urinary tract dysfunction
NR: Non response
PNR: Primary non response
ROC Curve: Receiver operating characteristic curve
SCI/D: Spinal cord injury/disease
SNR: Secondary non response
